# Refraining from seeking dental care among the Sámi in Sweden: a cross-sectional study

**DOI:** 10.1186/s12939-024-02305-1

**Published:** 2024-10-26

**Authors:** Negin Yekkalam, Christina Storm Mienna, Jon Petter Anders Stoor, Miguel San Sebastian

**Affiliations:** 1 Department of Odontology, Orofacial Pain and Jaw Function, Umeå University, Umeå, Sweden; 2 Várdduo – Centre for Sámi Research, Umeå University, Umeå, Sweden; 3 Department of Epidemiology and Global Health, Lávvuo-Research and Education for Sámi Health, Umeå University, Umeå, Sweden

**Keywords:** Sámi, Indigenous, Oral health, Refraining from dental care, Sápmi

## Abstract

**Background:**

While equity in health care is the core of the Swedish health system, social inequalities in accessing health care, particularly regarding dental care, exist. There is however no information on how the Sámi population is affected. This study aimed to assess the prevalence and risk factors for refraining from seeking dental care among the Sámi in Sweden.

**Methods:**

A Sámi sample was constructed from three pre-existing registers. Among the 9,249 invitations for individuals aged 18–84 years old, 3,779 answered the survey during February–May 2021. We first calculated frequencies and proportions of the independent variables in terms of socio-economic, socio-demographic, and cultural-related factors as well as the outcome, refraining from dental care. Then, we summarized the magnitude of the association between the independent variables and self-reported refrain from dental care with the prevalence difference (PD) using the 95% confidence interval (95% CI) for inferential purposes.

**Results:**

Overall, 17.5% of the participants refrained from seeking dental care despite self-reported need in the last three months, with almost the same proportion between men and women. Among the socio-demographic factors, being in the 30–44 years group (PD = 8.0; 95% CI: 3.59, 12.48), in the 45–64 group (PD = 7.3; 95% CI: 2.96, 11.61) and in the 65–84 group (PD = 5.4; 95% CI: 0.92, 9.78) as well as being divorced/widow-er (PD = 6.7; 95% CI: 2.73, 10.70) and unmarried (PD = 3.1; 95% CI: 0.23, 6.04) were statistically significantly associated with refraining from seeking dental care. Among the socio-economic variables, those in the middle-income quintile (PD = 5.3; 95% CI: 1.28, 9.35), in the poor (PD = 8.1; 95% CI: 3.64, 12.51) and poorest (PD = 8.0 95% CI: 3.48, 12.50) quintiles, and especially those experiencing economic stress once (PD = 9.2; 95% CI: 2.93, 15.48) and several times (PD = 26.5; 95% CI: 19.50, 33.43), were strongly associated with refraining.

**Conclusions:**

Approximately one in six of the Sámi participating in this study refrained from seeking dental care despite self-reported need in the last three months. Those who experienced economic difficulties were the most affected group. To achieve equity in dental health care in Sweden, policies removing economic barriers to access dental health care should be implemented.

## Background

The goal of any health care system should be to achieve equity in health and access to health care regardless of patients’ socio-economic position or other social circumstances [1]. However, health systems all over the world struggle to eliminate obstacles such as financial, geographical, and social barriers to accessing health care [2, 3].

Oral health is a crucial aspect of general health and is associated with the well-being of the entire person [4]. Thus, the development of oral health systems that are financially fair, allowing its access according to need, has been encouraged [5]. However, numerous studies have highlighted challenges regarding access to the dental health care services among socially disadvantaged groups, including ethnic minorities and socio-economically deprived populations [6–8]. Identifying these vulnerable groups in the local context is a crucial first step to addressing the problem. It is well-known that delays or lack of dental care may result in delayed diagnosis, untreated oral disease as well as compromised health status [9, 10].

The Swedish health care system is built on the concept of equity, aiming to achieve universal coverage through the guidance of an ethical platform [11] and by Swedish laws and policies [12]. Access to oral health care is free for children, adolescents, and young adults (currently up to 23 years of age), while adults pay a large part of the costs out of their own pocket. Studies suggest that the current structure is generating social inequalities in access to the dental health care services among the adult population, resulting in social differences in oral health [7]. In a 2012 survey among the Swedish general adult population, 10% of the respondents reported refraining from seeking dental care, with this being most common among the unemployed, those on a disability pension, or with long-lasting illness [13]. In another population-based study, foreign-born residents reported less access to dental care, despite greater needs, than Swedish-born residents [14].

The Sámi represent the sole indigenous group within the European Union, traditionally inhabiting the northern regions of Norway, Sweden, Finland, and north-western Russia [15]. Contemporary estimates indicate that there are about 20–40,000 Sámi residing in Sweden, although exact figures and demographic details are not available. Traditionally, the Sámi have engaged in activities such as hunting, fishing, foraging, reindeer herding, crafting handicrafts, and practising small-scale farming. However, in modern times, wage employment has become their predominant source of income [16].

Overall, oral health studies among the Sámi population are scarce [17–23]. In studies among the Sámi in Norway, a higher prevalence of poor oral health was reported in the Sámi-majority areas compared to those from Sámi minority areas [17, 18]. Also, a recent study showed that enrolment and integration of epidemiological data collection into the daily routine of public dental clinics and the creation of ethnic categories, such as Sámi, was feasible and provided clinical data with a satisfactory level of validity [19]. However, to our knowledge, literature identifying the social groups with difficulties in accessing dental health care is lacking. Therefore, the aim of this study was to assess the prevalence and risk factors for refraining from seeking dental care among the Sámi in Sweden.

## Methods

### Study design

This study is based on the data from the SámiHET survey, a population-based self-reported health study conducted among the Sámi population in Sweden. Statistics Sweden collected the data during February–May 2021, on behalf of the Department of Epidemiology and Global Health and the Sámidiggi (the Sámi parliament in Sweden). Information about the study was provided to participants in Sámi languages (North-, Lule-, and South Sámi) and Swedish, the questionnaire was available in Swedish. All participants provided their informed consent. A total of 9,249 individuals aged 18–84 were invited to participate after being identified through three administrative registers: (i) the Sámi parliament electoral roll; (ii) the reindeer mark register; and (iii) individuals receiving income from reindeer husbandry enterprises. In addition, Statistics Sweden linked registered data such as age, sex, educational level, and income to the survey through the personal identity number of the participants. The questionnaire was piloted among a sample of Sámi volunteers in November 2020. The study design followed the STROBE guidelines.

### Questionnaire

The 2021 SámiHET questionnaire comprised 81 questions related to 25 thematic areas, including oral health and health care.

### Outcome

The question “Have you, during the past three months, considered yourself in need of dental care, but refrained from seeking dental care?” was used to define self-reported refrain from seeking dental care. Respondents could choose one of the two options: “no” and “yes”. Those who answered affirmatively were asked for the reasons and if they answered that the ¨problem had disappeared¨ were classified as not refraining. This question is also used by the Public Health Agency of Sweden in their national surveys.

### Independent variables

We organized the independent variables into socio-demographic, socio-economic, and culture-related factors based on the literature [7, 11] and the available data.

Six variables were included as socio-demographic factors. Sex was dichotomized into men and women while sexual orientation identity into heterosexual and other. Age was divided into four groups (18–29, 30–44, 45–64 and 65–84 years) and civil status into three (married or cohabiting, divorced or widow-er, and unmarried). Self-reported living arrangement was split into living with others and alone. Place of residence included a rurality variable (large city, medium-sized city, small town/rural) and a regional variable: Norrbotten, Västerbotten, Jämtland Härjedalen, and other (representing the rest of the country). The rurality variable was based on the official classification of Sweden’s 290 municipalities [25], which considers each municipality’s population size, proximity to urban areas and commuting patterns to urban areas. We defined ‘large city’ as a municipality with > 200,000 inhabitants (Stockholm, Gothenburg, or Malmö), or a municipality which is close to a large city and is functionally connected to a large city by a high commuting frequency (> 40% of population); ‘medium-sized city’ includes a medium-sized municipality of > 50 000 inhabitants with at least 40,000 residing in the largest urban area, or a commuting or non-commuting municipality close to a medium-sized municipality; ‘small town or rural area’ are municipalities not meeting the criteria for either of the other two groups.

The three socio-economic variables included were: (i) the level of education, which we divided into low (compulsory, till 9 years), medium (secondary, 10–12 years) and high (postgraduate, > 12 years), as characterized by Statistics Sweden; (ii) individual disposable income, which we defined as the amount left for consumption or savings after taxes have been paid as well as all positive and negative transfers have been made (figures represent Swedish Krona-SEK; around 0.1 USD); income was divided into quintiles (quintile 1 being the richest) for each sex group; and (iii) we captured economic stress with the following question: “During the last 12 months, have you ever had difficulty in managing the regular expenses for food, rent, bills, etc.?” with ‘no,’ ‘yes, once,’ and ‘yes, more than once’ as possible options. We combined the affirmative answers in the analysis.

Finally, two questions were related to the Sámi cultural context: (i) if they belong or not to a Sámi reindeer herding community (RHC) (mountain, forest, concession, or none); and (ii) if they spoke a Sámi language fluently, or not.

### Data analysis

We first calculated frequencies and proportions of the independent variables and the outcome of the study sample. Then, we summarized the magnitude of association between the independent variables and self-reported refraining from seeking dental care with the prevalence difference (PD) using the 95% confidence interval (95% CI) for inferential purposes. First, we estimated crude regression models and included the statistically significant variables in the multivariable regression model. All analyses applied sampling weighting using the Stata 14 statistical software.

### Ethics

This study was conducted on behalf of the Sámi parliament in Sweden and researchers continuously consulted with the mandated members of the board of the parliament throughout the process. We informed the participants about the study and its aims before they consented to participate, and the Swedish Ethical Review Authority (Dnr 2020–04803, Ö 70-2020/3.1) approved the SámiHET study. In addition, the study followed the (Norwegian) Ethical Guidelines for Sámi Health Research (26). The study was performed in accordance with the ethical principles for medical research involving human subjects according to the World Medical Association Declaration of Helsinki.

## Results

Characteristics of the participants.

Among the 9,249 invitations, 3,779 answered the questionnaire corresponding to a participation rate of 40.9%. Among those, we excluded 121 individuals since they did not unequivocally identify themselves as Sámi, resulting in 3,658 individuals in the analytical sample.

Table 1 presents the characteristics of the participants in this study. The sex distribution was fairly equal (women 50.9% and men 49.2%), and regarding age, the 45–64 years old group (37.7%) was the largest and the youngest age group [18–24] the smallest (12.7%). Most of respondents lived with others (79.0%), and 46.0% were unmarried. More than two-thirds (67.0%) lived in a small town/rural area and almost one out of two were from the region of Norrbotten (48.9%).

Most of the participants had a medium level education (61.8%) and 3121 (85.8%) reported not to have experienced economic stress in the last 12 months. Approximately 60% of the participants did not belong to any Sámi RHC while around one-third (30.8%) belonged to a mountain and 11% to a forest reindeer herding community. Around one-quarter (22.9%) of participants were fluent in Sámi.

### Self-reported refraining from seeking dental care

Overall, 17.5% of the participants refrained from seeking dental care despite self-reported need in the last 3 months, with almost the same proportion between men (17.7%) and women (17.4%). We found a higher prevalence of refraining among the non-heterosexual group (20.1%), those who were 30–64 years-old (37.0%), those living alone (19.5%), were divorced/widow-er (23.6%), lived in a small town/rural area (18.1%) or resided in the region of Jämtland Hjärdalen (20.5%).

Those with medium level of education (19.0%), the poor and very poor income quintiles (21.0%), and those experiencing economic stress several times (43.0%) also reported higher levels of refraining from seeking dental care. Among the culture-related variables, those belonging to forest RHCs (21.4%) reported the highest prevalence of refraining.

In the crude model, the variables age, civil status, education, income, economic stress, and Forest Sámi RHC were statistically associated with refraining from seeking dental care. After adjustment, among the socio-demographic variables, being in the 30–44 years group (PD = 8.03; 95% CI: 3.59, 12.48), in the 45–64 group (PD = 7.28; 95% CI: 2.96, 11.61) and in the 65–84 group (PD = 5.35; 95% CI: 0.92, 9.78) as well as to be divorced/widow-er (PD = 6.72; 95% CI: 2.73, 10.70) and unmarried (PD = 3.13; 95% CI: 0.23, 6.04) remained statistically significantly associated with refraining from seeking dental care.

Among the socio-economic variables, those in the middle income quintile (PD = 5.31; 95% CI: 1.28, 9.35), in the poor (PD = 8.08; 95% CI: 3.64, 12.51) and poorest (PD = 7.99; 95% CI: 3.48, 12.50) quintiles, and especially those experiencing economic stress once (PD = 9.21; 95% CI: 2.93, 15.48), and several times (PD = 26.46; 95% CI: 19.50, 33.43) were strongly associated with refraining. While not statistically significant, those belonging to a forest RHC had a 4% points higher prevalence of refraining compared to those belonging to mountain reindeer herding communities (Table 2).

**Table 1:** Social characteristics of the total sample

	n (%)
**Socio-demographic factors**	
**Sex**	
Men	1798 (49.2)
Women	1860 (50.9)
**Sexual orientation identity**	
Heterosexual	3248 (91.2)
Non-heterosexual	312 (8.8)
**Age**	
18–29	463 (12.7)
30–44	861 (23.5)
45–64	1381 (37.7)
65–84	954 (26.1)
**Living arrangement**	
With others	2882 (78.8)
Alone	776 (21.2)
**Civil status**	
Married	1377 (37.6)
Divorced/Widow-er	586 (16.0)
Unmarried	1695 (46.3)
**Rurality**	
Large city	350 (9.6)
Medium-sized city	874 (23.9)
Small town/Rural area	2434 (66.5)
**Region**	
Norrbotten	1789 (48.9)
Västerbotten	845 (23.1)
Jämtland Härjedalen	268 (7.3)
Other	756 (20.7)
**Socio-economic factors**	
**Education**	
High	865 (23.7)
Medium	2258 (61.8)
Low	529 (14.5)
Mean income (sd) (in SEK)	266135 (245457)
**Economic stress**	
No	3121 (85.8)
Yes, once	249 (6.8)
Yes, several	266 (7.3)
**Culture-related factors**	
**Sámi reindeer herding community**	
Mountain	1112 (30.8)
Forest	390 (10.8)
Concession	50 (1.4)
None	2051 (59.6)
**Sámi language (fluently speaking)**	
Yes	815 (22.9)
No	2741 (77.1)

**Table 2:** Prevalence of refraining from seeking dental care, and the crude and adjusted regression estimations

	Refrain from dental care *n* (%)	Crude prevalence difference PD (95% CI)	Adjusted prevalence difference PD (95% CI)
**Socio-demographic factors**			
**Sex** Men Women	313 (17.7) 320 (17.4)	Ref -0.3 (-2.93, 2.30)	
**Sexual orientation identity** Heterosexual Non-heterosexual	562 (17.4) 61 (20.1)	Ref 1.3 (-1.18, 3.87)	
**Age** 18–29 30–44 45–64 65–84	63 (13.8) 158 (18.5) 255 (18.5) 157 (16.9)	Ref 4.7 (0.12, 9.32) 4.8 (0.56, 8.98) 3.2 (-1.10, 7.50)	Ref 8.0 (3.59, 12.48) 7.3 (2.96, 11.61) 5.4 (0.92, 9.78)
**Living arrangement** With others Alone	485 (16.9) 149 (19.5)	Ref 2.5 (-0.81, 5.89)	-
**Civil status** Married Divorced/Widow-er Unmarried	201 (14.7) 134 (23.6) 298(17.7)	Ref 8.9 (4.89, 13.01) 3.1 (0.32, 5.81)	Ref 6.7 (2.73, 10.70) 3.1 (0.23, 6.04)
**Rurality** Large city Medium-sized city Small town /rural area	56 (16.2) 143(16.5) 434 (18.1)	Ref 0.3 (-4.42, 5.05) 1.9 (-2.42, 6.17)	
**Region** Norrbotten Västerbotten Jämtland Härjedalen Other	320 (18.1) 133 (16.0) 55 (20.5) 125 (16.8)	Ref -2.1 (-5.32, 1.21) 2.4 (-3.19, 7.93) -1.3 (-4.65, 2.01)	
**Socio-economic factors**			
**Education** High Medium Low (9 year)	131 (15.2) 416 (18.9) 83 (16.2)	Ref 3.4 (0.59, 6.27) 1.0 (-3.23, 5.26)	Ref 0.1 (-2.83, 3.00) -3.9 (-8.49, 0.66)
**Income** Q1 very rich Q2 rich Q3 middle Q4 poor Q5 very poor	91 (12.8) 92 (13.0) 137 (19.1) 150 (21.4) 163 (20.9)	Ref 0.2 (-3.50, 3.82) 6.3 (2.29, 10.25) 8.5 (4.43, 12.62) 8.1 (4.08, 12.14)	Ref 0.3 (-3.89, 3.35) 5.3 (1.28, 9.35) 8.1 (3.64, 12.51) 8.0 (3.48, 12.50)
**Economic stress** No Yes, once Yes, several	456 (14.7) 63 (25.5) 112 (42.97)	Ref 10.7 (4.66, 16.83) 28.3 (21.48, 35.03)	Ref 9.2 (2.93, 15.48) 26.5 (19.50, 33.43)
**Culture-related factors**			
**Sámi community** Mountain Forest Concession None	180 (16.3) 83 (21.4) 5 (10.9) 355 (17.4)	Ref 5.1 (0.06, 10.21) -5.4 (-14.41,3.69) 1.1 (-1.75, 4.03)	Ref 4.1 (-0.78, 8.91) - 1.0 (-1.75, 3.77)
**Sámi language** Yes No	141 (17.5) 481 (17.6)	Ref 0.2 (-2.98, 3.29)	

* Adjusted for age, civil status, educations, income, economic stress and Sámi community

## Discussion

To the best of our knowledge, this is the first population-based study assessing refraining, despite self-reported need, from dental care among the Sámi indigenous population of Sweden. While demographic variables such as older age, non-being married and being divorced/widow-er were related to refraining, we observed the largest prevalence difference among those with self-reporting economic stress. More Sámi-specific indicators, including cultural, linguistic and urban-rural residence, did not seem to play a role. This suggests that reducing economic barriers to dental care would be the most important strategy for reducing dental care avoidance among the Sámi in Sweden. However, it should also be noted that other strategies - such as improving the availability of access to dental care in Sámi languages and improving cultural safety for Sámi patients accessing dental care - may still be valuable for improving other aspects of the Sámi experience of the dental health care system in Sweden. Such issues would be an appropriate focus for future studies.

In this study, the prevalence of refraining from seeking dental care was 17.5% among Sámi, which is similar (16.4%) to that reported by the adult general population in Sweden [27]. Among Sámi, the prevalence of refraining from seeking dental care was similar among men and women, which is also in line with the findings from the general population [28]. This differs however from a Swedish study, which observed a higher refrain among women, due to lower income and poorer financial resources, compared to men [7].

It was not surprising that the youngest group [18–29] had the lowest prevalence of refraining given that dental care is free up to 23 years of age in Sweden. Nevertheless, previous national studies have not observed this pattern, with older ages reporting lower prevalence of refraining [28, 29]. However, in our study, there was still a considerable number of young Sámi aged 18–29 who were refraining (13.8%), particularly in the 18–23 age group (15.7%), highlighting a complex pattern of oral health care-seeking behaviour that requires further investigation.

In our study, those who were divorced/widow-er and unmarried refrained more from dental care compared to those who were married. Some have postulated that a beneficial effect of marriage works by offering more social support and control of health behaviours between partners, particularly among men [30], and evidence exists linking a lack of social support with refraining from seeking dental care [28, 31].

The association between low income and economic stress and refraining from seeking dental care was expected since numerous studies, both from Sweden [7, 28, 31, 32] and internationally, have observed this [33]. Previous studies from Sweden have proposed the cost of dental treatment as one main barrier to accessing oral health care [7, 34]. In a population survey among 18–84-year-olds in Sweden, 10% reported that they refrained from seeking dental care due to financial reasons [29]. In another study among the adult population from Southern Sweden, 18% of the participants reported the cost for dental care as one of the main factors for refraining from seeking dental care [35]. In turn, refraining can result in poorer dental health as previous studies have shown [7, 29, 36].

While the literature has reported geographical distance to dental health care and cultural factors as important risk factors for refraining from seeking dental care [36–38], this was not the case in our study. Further research may investigate if other geographic or cultural factors may be more related.

### Methodological considerations

This study is based on a large sample of Sámi participants. The applied weighting in the analysis together with the use of several registered-based variables further strengthen the external and internal validity of the findings. However, since the Sámi demography remains unknown, it is not possible to extend these findings to the entire Sámi population in Sweden. Further, the moderate participation rate might indicate a certain selection bias, and thus affect the generalizability of the findings to the entire population. However, the participation rate was similar to the Swedish national public health survey, conducted simultaneously [40]. Given the self-reported nature of the survey, participants might have provided inaccurate or incomplete information in some of the variables, leading to recall and reporting bias. Nevertheless, the impact of these biases on the estimated figures is not possible to assess. The fact that most of the sample came from Norrbotten region could have influenced the findings. In addition, the lack of association with rurality and cultural factors might also be indicating the crudeness and inappropriateness of these variables to capture refraining from dental health care, which future studies should take into consideration.

## Conclusion

Approximately one in six of the Sámi participating in this study refrained from seeking dental care despite self-reported need in the last three months, with almost the same proportion between men and women. Those who experienced economic difficulties were the most affected group. Based on our findings, the recommended strategy for achieving equity in dental health care among Sámi in Sweden is to implement policies that remove economic barriers to access to dental care. In this way, not only can treatment be provided, but also the prevention of future complications can be avoided.

## Data Availability

The dataset supporting the conclusions of this article is not publicly available due to its sensitive nature, including information on health and ethnicity. For more information regarding data availability, please contact MSS, the last author of this manuscript.

## Abbreviations

CI: Confidence Interval
PD: Prevalence Difference
RHC: Sámi reindeer herding community
