# Correction: Refraining from seeking dental care among the Sámi in Sweden: a cross-sectional study

**DOI:** 10.1186/s12939-024-02329-7

**Published:** 2024-11-14

**Authors:** Negin Yekkalam, Christina Storm Mienna, Jon Petter Anders Stoor, Miguel San Sebastian

**Affiliations:** 1 Department of Odontology, Orofacial Pain and Jaw Function, Umeå University, Umeå, Sweden; 2 Várdduo – Centre for Sámi Research, Umeå University, Umeå, Sweden; 3 Department of Epidemiology and Global Health, Lávvuo-Research and Education for Sámi Health, Umeå University, Umeå, Sweden

**Correction: Int J Equity Health** **23, 222 (2024)**

10.1186/s12939-024-02305-1

In the original article [1], Christina Storm Mienna was incorrectly denoted as co-corresponding author, but the sole corresponding author should have been Negin Yekkalam.

The original article [1] has been updated.
